# Association between platelet-lymphocyte ratio and 90-day mortality in patients with intracerebral hemorrhage: data from the MIMIC-III database

**DOI:** 10.3389/fneur.2023.1234252

**Published:** 2023-10-09

**Authors:** Min Yuan, Zhilong Xiao, Huangyan Zhou, Anxia Fu, Zhimin Pei

**Affiliations:** ^1^Graduate School, Nanchang University, Nanchang, China; ^2^Department of Neurology, Jiangxi Provincial People's Hospital, The First Affiliated Hospital of Nanchang Medical College, Nanchang, China; ^3^Department of Neurology, The Third Hospital of Nanchang, Nanchang, China; ^4^Department of Blood Transfusion, Jiangxi Cancer Hospital, The Second Affiliated Hospital of Nanchang Medical College, Jiangxi Clinical Research Center for Cancer, Nanchang, China; ^5^The Second People's Hospital of Nanchang County, Nanchang, China

**Keywords:** intracerebral hemorrhage, platelet-lymphocyte ratio, mortality, retrospective cohort study, MIMIC database

## Abstract

**Background:**

Recent evidence suggested that platelet-lymphocyte ratio (PLR) may play a role in the pathophysiology of intracerebral hemorrhage (ICH), but the results are controversial. This study aimed to explore the relationship between PLR and mortality in patients with ICH.

**Methods:**

All data were extracted from the Medical Information Mart for Intensive Care (MIMIC) III database. The study outcome was 90-day mortality. Multivariable Cox regression analyses were used to calculate the adjusted hazard ratio (HR) with a 95% confidence interval (CI), and curve-fitting (restricted cubic spline) was used to assess the non-linear relationship.

**Results:**

Of 1,442 patients, 1,043 patients with ICH were included. The overall 90-day mortality was 29.8% (311/1,043). When PLR was assessed in quartiles, the risk of 90-day mortality for ICH was lowest for quartile 2 (120.9 to <189.8: adjusted HR, 0.67; 95% CI: 0.48–0.93; *P* = 0.016), compared with those in quartile 1 (<120.9**)**. Consistently in the threshold analysis, for every 1 unit increase in PLR, there was a 0.6% decrease in the risk of 90-day mortality for ICH (adjusted HR, 0.994; 95% CI: 0.988–0.999) in those with PLR <145.54, and a 0.2% increase in 90-day mortality (adjusted HR, 1.002; 95% CI: 1.000–1.003) in participants with PLR ≥145.54.

**Conclusion:**

There was a non-linear relationship between PLR and 90-day mortality for patients with ICH, with an inflection point at 145.54 and a minimal risk at 120.9 to <189.8 of PLR.

## 1. Introduction

Intracerebral hemorrhage (ICH) is the most common hemorrhage stroke subtype, leading to high mortality and morbidity (1). The global incidence of ICH is increasing with an aging population, accounting for 10–15% of all strokes in Western countries and 20–30% in Asia (2). A significant number of ICH patients required long-term hospitalization and rehabilitation (3). Thus, ICH incurs a severe burden on patients, caregivers, family members, and society. Due to ICH's extremely high mortality and morbidity rates, early identification of high-risk patients is of great significance for clinical treatment and family care. Various clinical and radiographic factors, including hemorrhagic volume, hematoma expansion, systolic blood pressure, coagulopathy, hyperglycemia, serum fibrinogen level, hematoma volume, and spot and swirl sign perihematomal edema, have served as predictors of poor outcome (4–6). Unfortunately, the prognosis after ICU has not improved in recent years (7).

Increasing evidence has proven that inflammation plays a crucial role in secondary brain injury induced by ICH, and various stimuli cause inflammation to progress after ICH (8–10). Hematoma components initiate an inflammatory response characterized by activating microglia and inflammatory-related cells, such as neutrophils, monocytes, and lymphocytes, and the release of pro-inflammatory cytokines and chemokines attracts peripheral inflammatory infiltration (11). Similarly, platelets are the key contributors to the immunomodulatory and inflammatory processes and interact with other inflammatory cells (12). These inflammatory cascade reactions could cause secondary brain injury (13). Previous studies have shown that platelet-lymphocyte ratio (PLR) is closely associated with the severity and prognosis of inflammation-associated diseases, including cardiac-cerebral vascular disease (14–18), chronic autoimmune disease (19), and malignant tumor (20, 21). Some studies showed that higher PLR was associated with the Glasgow Coma Scale (GCS) at hospital discharge but not with long-term neurological outcomes in patients with ICH (22). Zou et al. (23) reported that high PLR is negatively related to the overall survival and prognosis of patients with cerebral hemorrhage. The relationship between PLR and ICU mortality is still controversial. Nowadays, an easily acquired and valuable measure that can predict ICH patients' outcomes has become a research hotspot. Therefore, we performed this study to explore the relationship between PLR and mortality in patients with ICH.

## 2. Methods

### 2.1. Database source

All the data in the current study were extracted from an extensive, freely available database, Medical Information Mart for Intensive Care (MIMIC) III (version 1.4), comprising more than 40,000 patients who stayed in the ICU of the Beth Israel Deaconess Medical Center (Boston, MA, USA) from 2001 to 2012 (24, 25). The database was published by the Massachusetts Institute of Technology (Cambridge, MA, USA), with approval by the Massachusetts Institute of Technology and Beth Israel Deaconess Medical Center's Institutional Review Boards. Two authors gained permission to access the database by completing an online training course at the National Institutes of Health (certification number: 27658233 for ZX and 46868266 for MY). They were responsible for the original data collection and the data extraction required in this study. PostgreSQL Tools version 12.5 was used to extract data from the database. Our research has also been approved and agreed upon by the Ethics Committee of Jiangxi Provincial People's Hospital, and the ethics number is 2022-025.

### 2.2. Study population and stratification method

Patients older than 18 years and diagnosed with ICH were selected for this study. Patients were excluded if they met the following criteria: (1) the patients had no data of lymphocytes within the first 24 h of admission and had >15% missing data; (2) the patients were diagnosed with hemorrhage due to brain trauma, brain tumor, or meningioma, and bleeding due to vascular abnormalities or arterial aneurysm; (3) the patients were diagnosed with hematological diseases such as leukemia or lymphoma. The following information was extracted: age, sex, mean arterial pressure, ethnicity, weight, alcohol drinking, smoking status, and comorbidities (atrial fibrillation, diabetes, hypertension, chronic obstructive pulmonary disease (COPD), coronary, heart failure, and hyperlipemia), as well as glucose, Ca+, Na+, K+, serum creatinine, serum urea nitrogen, white blood cell, blood platelet, blood lymphocyte, activated partial thromboplastin time (APTT), PT, location of ICH, statin user, anticoagulant, antiplatelet agents, length of hospital stay and ICU stay, GCS score, sequential organ failure assessment (SOFA) score at admission, blood transfusion, sepsis, and the use of mechanical ventilation during hospitalization. Statins include atorvastatin, lovastatin, pravastatin, rosuvastatin, and simvastatin. Anticoagulant therapy refers to patients who use warfarin, heparin, and new anticoagulants. Antiplatelet agents refer to aspirin and clopidogrel. The PLR was calculated using the platelet count divided by the lymphocyte count, equal to the white blood cell count multiplied by the lymphocyte percentage divided by 100. Only the values of blood lymphocytes and platelets examined for the first time were simultaneously used in the calculation. Only the record of the first admission should be considered for patients who have been admitted repeatedly.

### 2.3. Outcome

The outcome was 90-day mortality.

### 2.4. Statistical analysis

Continuous variables are presented as mean ± standard deviation (SD) or median (interquartile ranges), as the case may be. Student's *t*-test, Wilcoxon rank-sum test, or Kruskal–Wallis test were used as appropriate. The X^2^ test was used to present categorical variables as a percentage. Multivariable Cox regression analyses assessed the independent association between PLR and 90-day mortality. An extended Cox model approach was used for different covariates-adjusted models. We constructed three models: Model 1 was adjusted only for age and sex; model 2 was additionally adjusted for COPD, heart failure, and hyperlipemia; and model 3 was additionally adjusted for ethnicity, blood glucose, serum creatinine, serum urea nitrogen, serum calcium, APTT, alcohol drinker, location of ICH, statin user, and antiplatelet agents. In order to evaluate the confusion, the covariates are input into the Cox regression model in the basic model or eliminated in the complete model, and the regression coefficients are compared. We mainly include covariates based on clinical experience and covariates that change the initial regression coefficient by more than 10%.

In addition, restricted cubic spline (RCS) regression was performed with four knots at the 5th, 35th, 65th, and 95th percentiles of PLR to assess linearity and examine the dose–response curve between PLR and ICH mortality after adjusting variables in model 3.

Threshold analysis of the association of PLR with the study outcome was conducted with a 2-piecewise Cox regression model using a smoothing function. The threshold level (i.e., inflection point) was determined using a likelihood-ratio test and bootstrap resampling methods (26). Additional exploratory analyses, including the following variables: age (<65 vs. ≥65 years), sex (male vs. female), atrial fibrillation (no vs. yes), COPD (no vs. yes), hyperlipidemia (no vs. yes), statin user (no vs. yes), and possible modifications on the association of PLR and 90-day mortality for patients with ICH were evaluated using stratified analyses and interaction testing.

A two-tailed test was performed, and *P* < 0.05 was considered statistically significant. The statistical software packages R 3.6.3 (http://www.R-project.org, The R Foundation) and Free Statistics software version 1.71 were used for all data analyses.

## 3. Results

### 3.1. Patient characteristics and outcome

**Figure 1:**
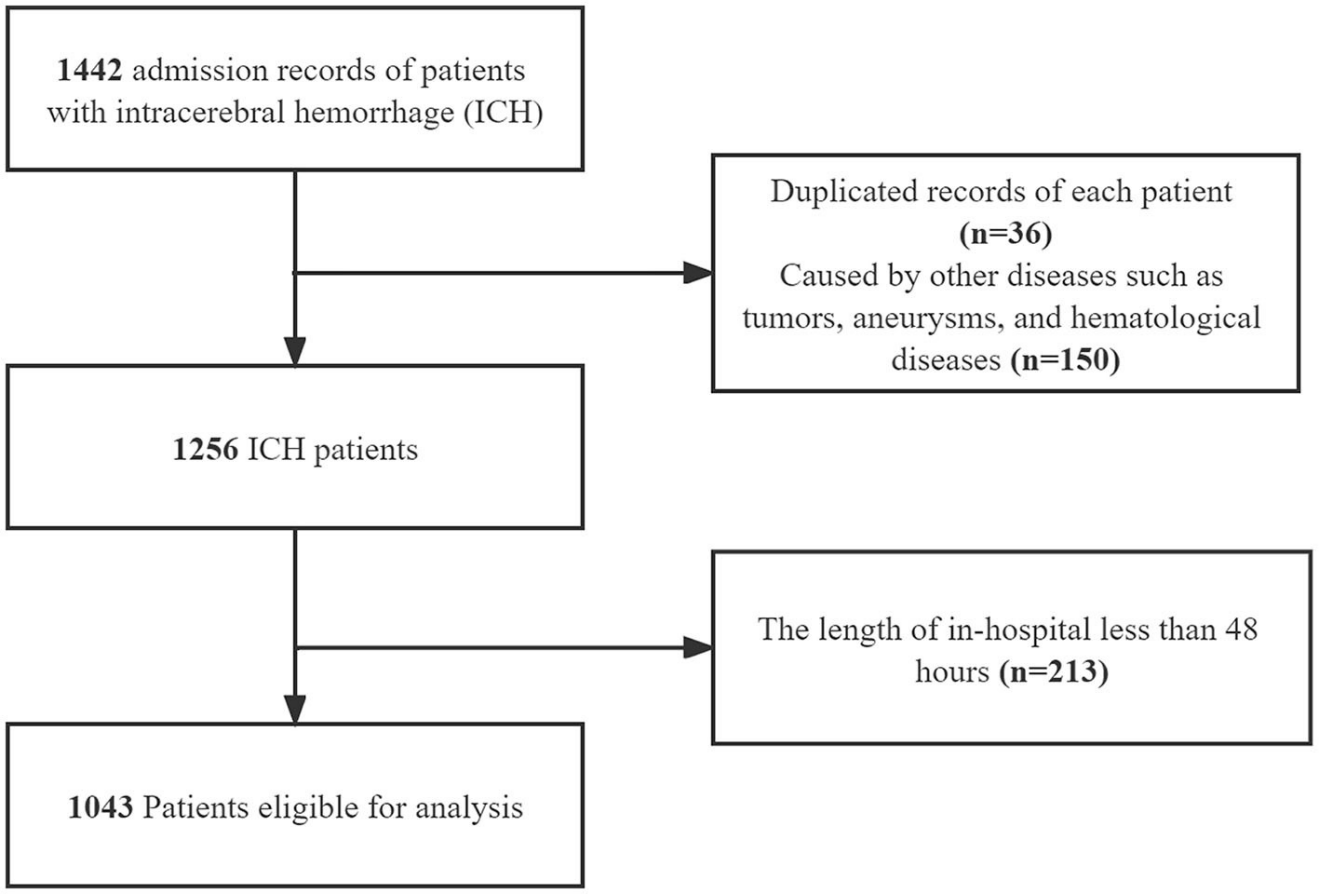
The flowchart of the study.

Of a total of 1,442 patients, 1,043 were included. We excluded 36 patients because of duplicated records, and another 150 patients with other diseases such as tumors, aneurysms, and hematological disorders were also excluded. In addition, we excluded 213 patients because the in-hospital length was <48 h. Finally, 1,043 patients were eligible for our analysis. The flowchart of the study patients' selection is presented in Figure 1.

The baseline characteristics of all participants are listed in Table 1. The age of all participants was 68.2 ± 15.1 years, 570 (54.7%) were men, 745 (71.4%) were white individuals, and 298 (28.6%) were non-white individuals. PLR is divided into four groups according to quartile [Q1 (<120.9), Q2 (120.9 to <189.8), Q3 (189.8 to <296.5), and Q4 (≥296.5)]. The median PLR was 189.8 (120.8, 295.6). The overall 90-day mortality was 29.8% (311/1043). Participants with higher PLR were more likely to be men, more white individuals, and less black individuals; these people were less likely to smoke and drink, and fewer patients had atrial fibrillation, diabetes, hypertension, hyperlipidemia, and heart failure, but they had a higher incidence of COPD and coronary heart disease. At the same time, patients with more elevated PLR had lower serum sodium, potassium, and calcium levels.

**Table 1:** Baseline characteristics of participants.

**Covariates**	**Total**	**Q1 (<120.9)**	**Q2 (120.9 to <189.8)**	**Q3 (189.8 to <296.5)**	**Q4 (≥296.5)**	***P*-value**
	**(*****n** =* **1,043)**	**(*****n** =* **261)**	**(*****n** =* **260)**	**(*****n** =* **261)**	**(*****n** =* **261)**	
Age (years)	68.2 ± 15.1	67.4 ± 15.7	69.0 ± 14.5	69.9 ± 14.3	66.7 ± 15.5	0.070
Male, sex, *n* (%)	570 (54.7)	156 (59.8)	146 (56.2)	136 (52.1)	132 (50.6)	0.143
MAP (mmHg)	85.5 ± 10.8	85.3 ± 10.3	86.0 ± 10.7	85.8 ± 10.2	85.1 ± 11.9	0.734
**Ethnicity**, ***n*** **(%)**						**0.101**
White	745 (71.4)	168 (64.4)	188 (72.3)	195 (74.7)	194 (74.3)	
Black	80 (7.7)	29 (11.1)	17 (6.5)	16 (6.1)	18 (6.9)	
Others	218 (20.9)	64 (24.5)	55 (21.2)	50 (19.2)	49 (18.8)	
Weight (kg)	76.9 ± 18.4	78.7 ± 17.6	78.5 ± 19.1	76.9 ± 19.5	73.5 ± 17.2	0.004
Alcohol drinker, *n* (%)	85 (8.1)	24 (9.2)	19 (7.3)	26 (10)	16 (6.1)	0.363
Smoker, *n* (%)	119 (11.4)	32 (12.3)	28 (10.8)	38 (14.6)	21 (8)	0.123
**Comorbidities**, ***n*** **(%)**						
Atrial fibrillation	266 (25.5)	61 (23.4)	68 (26.2)	79 (30.3)	58 (22.2)	0.152
Diabetes	241 (23.1)	64 (24.5)	71 (27.3)	57 (21.8)	49 (18.8)	0.118
Hypertension	806 (77.3)	196 (75.1)	212 (81.5)	211 (80.8)	187 (71.6)	0.019
COPD	93 (8.9)	23 (8.8)	17 (6.5)	29 (11.1)	24 (9.2)	0.336
Coronary	180 (17.3)	42 (16.1)	47 (18.1)	48 (18.4)	43 (16.5)	0.869
Heart failure	146 (14.0)	38 (14.6)	36 (13.8)	40 (15.3)	32 (12.3)	0.775
Hyperlipemia	307 (29.4)	79 (30.3)	79 (30.4)	92 (35.2)	57 (21.8)	0.
**Laboratory parameters**						
Blood glucose (mg/dl)	133.4 ± 41.0	134.8 ± 39.9	136.1 ± 42.7	130.0 ± 41.0	132.6 ± 40.3	0.340
Serum calcium (mg/dl)	8.7 ± 0.7	8.8 ± 0.7	8.8 ± 0.7	8.7 ± 0.7	8.7 ± 0.7	0.698
Serum sodium (mg/dl)	139.5 ± 4.7	140.3 ± 4.9	139.2 ± 4.2	139.2 ± 4.8	139.4 ± 4.8	0.017
Serum potassium (mEq/L)	3.9 ± 0.5	3.9 ± 0.5	3.9 ± 0.6	3.9 ± 0.5	3.9 ± 0.5	0.765
Serum creatinine (mg/dl)	0.8 (0.7, 1.1)	0.9 (0.7, 1.2)	0.8 (0.7, 1.2)	0.8 (0.7, 1.1)	0.8 (0.6, 1.0)	<0.001
Serum urea nitrogen (mg/dl)	19.0 (13.0, 26.0)	20.0 (14.0, 26.0)	18.0 (13.0, 25.0)	18.0 (13.0, 25.0)	19.0 (14.0, 27.0)	0.507
White blood cell (K/ul)	10.7 ± 4.1	12.0 ± 4.6	10.5 ± 3.5	10.2 ± 3.7	10.2 ± 4.3	<0.001
Blood platelet (K/ul)	251.5 ± 107.9	186.8 ± 75.1	236.3 ± 83.1	264.9 ± 100.6	318.2 ± 122.2	<0.001
Blood lymphocyte (%)	13.2 (8.4, 16.9)	19.2 (14.0, 27.4)	14.6 (12.8, 18.4)	11.6 (8.6, 14.6)	6.7 (4.6, 9.5)	<0.001
APTT (s)	29.0 ± 9.8	29.3 ± 10.0	28.6 ± 9.8	29.2 ± 9.5	29.0 ± 10.1	0.851
PT (s)	14.0 ± 3.3	14.3 ± 3.6	13.6 ± 2.6	14.0 ± 3.3	14.1 ± 3.5	0.142
**Location of ICH**, ***n*** **(%)**						**0.267**
Cerebral hemisphere	873 (83.7)	222 (85.1)	222 (85.4)	216 (82.8)	213 (81.6)	
Cerebellum	86 (8.2)	16 (6.1)	23 (8.8)	17 (6.5)	30 (11.5)	
Brainstem	40 (3.8)	11 (4.2)	6 (2.3)	14 (5.4)	9 (3.4)	
Ventricle	44 (4.2)	12 (4.6)	9 (3.5)	14 (5.4)	9 (3.4)	
**Drugs**, ***n*** **(%)**						
Statin user	362 (34.7)	81 (31)	95 (36.5)	102 (39.1)	84 (32.2)	0.181
Anticoagulant	153 (14.7)	36 (13.8)	37 (14.2)	46 (17.6)	34 (13)	0.46
Antiplatelet agents	155 (14.9)	40 (15.3)	41 (15.8)	44 (16.9)	30 (11.5)	0.337
Transfusion, *n* (%)	255 (24.4)	72 (27.6)	62 (23.8)	55 (21.1)	66 (25.3)	0.369
Sepsis, *n* (%)	325 (31.2)	79 (30.3)	66 (25.4)	69 (26.4)	111 (42.5)	<0.001
Mechanical ventilation, *n* (%)	574 (55.0)	142 (54.4)	129 (49.6)	142 (54.4)	161 (61.7)	0.050
GCS score	11.5 ± 3.7	11.2 ± 3.9	12.0 ± 3.5	11.7 ± 3.5	11.2 ± 3.7	0.034
SOFA score	3.0 (2.0, 4.0)	3.0 (2.0, 5.0)	3.0 (1.0, 4.0)	2.0 (2.0, 4.0)	3.0 (1.0, 4.0)	0.028
PLR	189.8 (120.8, 295.6)	90.3 (70.6, 107.4)	151.0 (135.8, 169.3)	231.1 (209.6, 261.7)	423.4 (353.0, 588.5)	<0.001
Los hospital (day)	12.0 ± 12.4	11.9 ± 12.7	10.6 ± 13.5	11.9 ± 10.6	13.5 ± 12.3	0.068
Los ICU (day)	5.7 ± 6.4	5.5 ± 6.4	4.8 ± 6.0	5.4 ± 5.5	6.8 ± 7.3	0.003
90-day mortality, *n* (%)	311 (29.8)	88 (33.7)	66 (25.4)	71 (27.2)	86 (33)	0.093

GCS, Glasgow Coma Scale; SOFA, sequential organ failure assessment; PLR, platelet-lymphocyte count ratio; COPD, chronic obstructive pulmonary disease; APTT, activated partial thromboplastin time; PT, prothrombin time; MAP, mean arterial pressure; Los, length of stay. Q1–Q4 represent the results of the quartile classification of PLR, respectively.

### 3.2. Relationship between PLR and 90-day mortality

In the extended multivariable Cox models, when PLR was assessed in quartiles and compared with Q1 (<120.9), we observed that the hazard ratios (HRs) of PLR and 90-day mortality for ICH in Q2 (120.9 to <189.8), Q3 (189.8 to <296.5), and Q4 (≥296.5) were 0.67 (95% CI: 0.48–0.93, *P* = 0.016), 0.71 (95% CI: 0.52–0.98, *P* = 0.035), and 0.96 (95% CI: 0.71–1.31, *P* = 0.788) after adjustment for all covariates (Table 2), respectively.

**Table 2:** Association between PLR and 90-day mortality in multiple regression model.

**Variable**	**n.total**	**n.event (%)**	**Crude**	***P*-value**	**Model 1**	***P*-value**	**Model 2**	***P*-value**	**Model 3**	***P*-value**
			**HR (95% CI)**		**HR (95% CI)**		**HR (95% CI)**		**HR (95% CI)**	
**Quartiles**										
Q1 (<120.9)	261	88 (33.7)	1 (Ref)		1 (Ref)		1 (Ref)		1 (Ref)	
Q2 (120.9 to <189.8)	260	66 (25.4)	0.70 (0.51–0.96)	0.026	0.67 (0.48–0.92)	0.012	0.66 (0.48–0.91)	0.011	0.67 (0.48–0.93)	0.016
Q3 (189.8 to <296.5)	261	71 (27.2)	0.74 (0.54–1.02)	0.063	0.68 (0.5–0.93)	0.016	0.68 (0.49–0.93)	0.015	0.71 (0.52–0.98)	0.035
Q4 (≥296.5)	261	86 (33)	0.94 (0.70–1.27)	0.688	0.95 (0.71–1.28)	0.746	0.93 (0.69–1.26)	0.64	0.96 (0.70–1.31)	0.788
*P* for trend. test				0.795		0.769		0.682		0.874
**Categories**										
Q1 (<120.9)	261	88 (33.7)	1.39 (1.06–1.82)	0.016	1.49 (1.14–1.95)	0.004	1.50 (1.14–1.96)	0.003	1.45 (1.10–1.91)	0.008
Q2–Q3 (120.9 to <296.5)	521	137 (26.3)	1 (Ref)		1 (Ref)		1 (Ref)		1 (Ref)	
Q4 (≥296.5)	261	86 (33)	1.31 (1.00–1.71)	0.052	1.42 (1.08–1.86)	0.012	1.39 (1.06–1.83)	0.017	1.39 (1.05–1.83)	0.02
*P* for trend test				0.693		0.740		0.639		0.787

Model 1: Adjusted for sex and age. Model 2: Adjusted for the variables in model 1 plus COPD, heart failure, and hyperlipemia. Model 3: Adjusted for the variables in model 2 plus ethnicity, blood glucose, serum creatinine, serum urea nitrogen, serum calcium, APTT, alcohol drinker, location of ICH, statin user, and antiplatelet agents.

**Figure 2:**
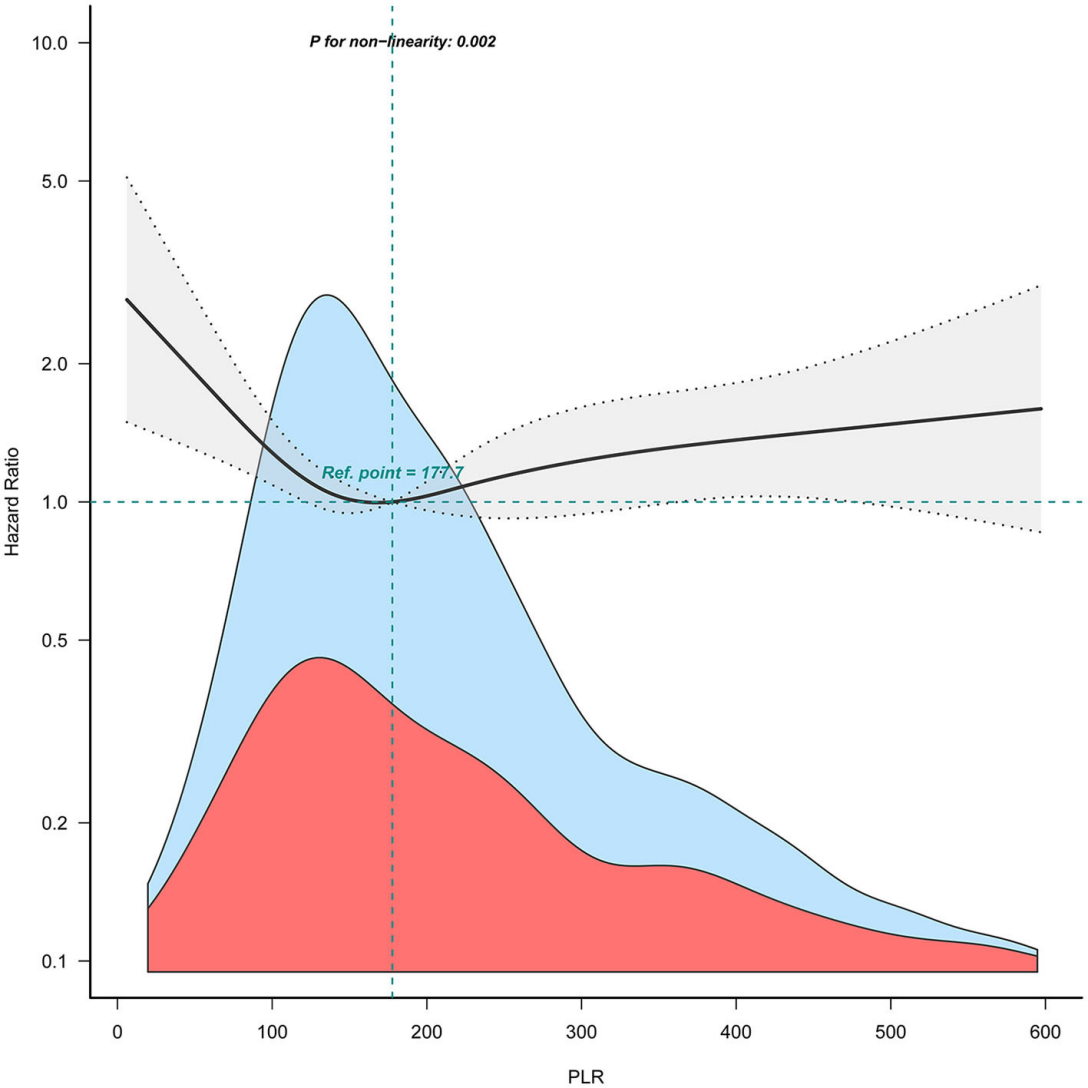
Relation of PLR with risk of 90-day mortality for patients with ICH. The shaded area indicates 95% confidence intervals for adjusted hazard ratios (HR). The model was adjusted for sex, age, COPD, heart failure, hyperlipemia, ethnicity, blood glucose, serum creatinine, serum urea nitrogen, serum calcium, APTT, alcohol drinker, location of ICH, statin user, and antiplatelet agents.

When combining quartiles in further exploratory analysis, a significantly higher risk of 90-day mortality was also found among participants in Q1 (<120.9: adjusted HR, 1.45; 95% CI: 1.10–1.91; *P* = 0.008) and in Q4 (≥296.5: adjusted HR, 1.39; 95% CI: 1.05–1.83; *P* = 0.02) compared with those in Q2–Q3 (120.9 to <296.5 model 3, Table 2). Accordingly, the association between PLR and 90-day mortality for patients with ICH followed a non-linear relationship (*P* for non-linearity = 0.002) in RCS (Figure 2).

Consistently in the threshold analysis, for every 1 unit increase in PLR, there was a 0.6% decrease in the risk of 90-day mortality for ICH (adjusted HR, 0.994; 95% CI: 0.988–0.999) in those with PLR <145.54, and a 0.2% increase in 90-day mortality (adjusted HR, 1.002; 95% CI: 1.000–1.003) in participants with PLR ≥ 145.54 (Table 3).

**Table 3:** Threshold analyses of PLR on 90-day mortality for patients with ICH using 2-piecewise regression models.

**PLR**	**Crude model**		**PLR**	**Adjusted model** ^**a**^	
	**HR (95% CI)**	***P*** **-value**		**HR (95% CI)**	***P*** **-value**
<156.23	0.994 (0.989–0.999)	0.013	<145.54	0.994 (0.988–0.999)	0.026
≥156.23	1.002 (1.000–1.003)	0.019	≥145.54	1.002 (1.000–1.003)	0.037

HR, hazard ratio. ^a^ Adjusted for sex, age, COPD, heart failure, hyperlipemia, ethnicity, blood glucose, serum creatinine, serum urea nitrogen, serum calcium, APTT, alcohol drinker, location of ICH, statin user, and antiplatelet agents.

To test the robustness of the association, we further performed a series of sensitivity analyses. First, further adjustments for hypertension history, mean arterial pressure, and anticoagulant use did not substantially change the results (eTable 1). Second, further adjustments for the blood transfusion, sepsis, and the use of mechanical ventilation also did not materially alter the findings (eTable 1).

### 3.3. Stratified analyses by potential effect modifiers

**Figure 3:**
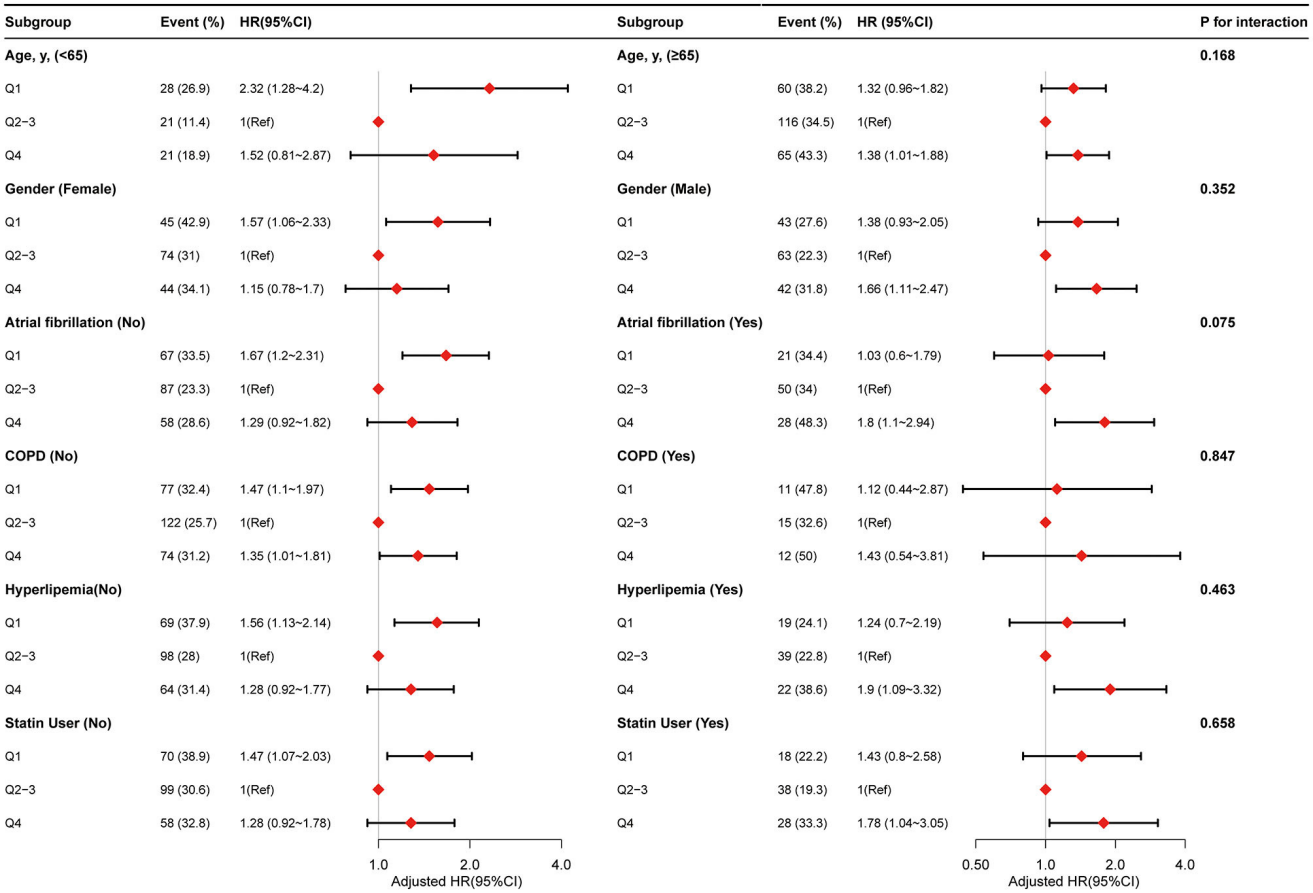
Stratified analyses by potential modifiers of the association between PLR with risk of 90-day mortality.

We performed further stratified analyses to assess the association between PLR (Q1 vs. Q2–Q3 vs. Q4) and the risk of 90-day mortality for patients with ICH in various subgroups (Figure 3). Overall, the non-linear relationship between PLR and 90-day mortality for patients with ICH was observed in the subgroups. None of the variables, including age (<65 vs. ≥65 years), sex (female vs. male), atrial fibrillation (no vs. yes), COPD (no vs. yes), hyperlipidemia (no vs. yes), and statin user (no vs. yes), significantly modified the association between PLR and 90-day mortality for patients with ICH (Figure 3). Furthermore, we did not observe significant interaction in the subgroups (*P*-value for interaction >0.05 for all).

## 4. Discussion

In this relatively large-scale cohort study, we found a non-linear relationship between PLR and 90-day mortality for patients with ICH, with an inflection point at 145.54 and minimal risk at 120.9–189.8 of PLR.

Previous studies have shown that PLR is a valuable inflammation biomarker in various diseases (27–30), and some trials have also assessed the relationship between PLR and ICH outcomes. Still, these trials reported inconsistent results, and the relationship between PLR and ICH mortality has not yet been elucidated. Zhang et al. (22) conducted a retrospective study and found that the PLR value >100 or the third and fourth quartiles of the PLR value on ICU admission was associated with worse GCS scores in 183 ICH patients but not with long-term neurological outcomes in patients with ICH. Zou et al. (23) reported that high PLR is negatively associated with the overall survival and prognosis of patients with cerebral hemorrhage. In addition, Tao et al. (31) analyzed 247 patients with subarachnoid hemorrhage and found that increased PLR was associated with a worse 3-month functional outcome after the onset. A study that included 57 patients with acute ischemic strokes who underwent mechanical thrombectomy revealed that higher PLR values were associated with poor prognoses (32). These studies show that the association between PLR and ICH is still uncertain. It is worth noting that these studies do not have detailed information about PLR thresholds. Although it is reported that different PLR values may have other outcome effects, the relationship between PLR and ICH has not been thoroughly studied. Our study provides an opportunity to assess a non-linear relationship between PLR and ICH mortality risk and comprehensively adjusts many known covariates and a series of subgroup analyses. We also used a comprehensive multi-model regression analysis to adjust for potential confounders. We found a stable relationship between PLR and ICH mortality risk, all of which confirmed that our results were very stable. The study found that PLR is a new index that can predict the 90-day mortality of ICH, with an inflection point at 145.54 and minimal risk at 120.9–189.8 of PLR. In future clinical work, we can predict the 90-day mortality rate of ICH through the calculation of PLR, and we can also compare the difference in 90-day mortality of different patients by comparing the PLR values of patients. In addition, we may reduce the 90-day mortality of patients with ICH by intervening in the number of platelets and lymphocytes; more importantly, platelets and lymphocytes can be very easy to obtain and affordable in clinics, which is of great significance for the work of clinicians. We hope our findings will inspire clinicians, which can provide evidence that can be considered for the treatment of patients and further in-depth research.

Our research provides some new insights. First, among participants whose PLR was <145.54, the risk of 90-day death in ICH patients decreased with the increase in PRL. PLR is simple to test and easy to obtain in the clinic and is related to inflammation and immune response. The immune-inflammatory response after cerebral hemorrhage is a complex pathophysiological process. Brain edema and inflammatory reactions are the key factors that promote ICH development. Inflammation was previously thought to be the result of the stress response. It is reported that the hyperacute inflammatory reaction of cerebral hemorrhage plays a protective role in promoting hemostasis and reducing hematoma expansion (33). Cerebral hemorrhage involves a series of pathophysiological processes. Inflammation around the hematoma has an essential effect on nerve injury, characterized by the aggregation of neutrophils, macrophages, monocytes, and microglia activation (34). To sum up, a reasonable biological explanation for the observed relationship between PLR and ICH mortality may be that PLR can protect ICH patients by regulating the inflammatory response after ICH. However, this mechanism needs to be further studied.

Second, among participants with a PLR of 145.54 or higher, the risk of ICH 90-day mortality increased with the increase in PLR. Experimental studies have shown that with the progress of the course of ICH, a series of complex inflammatory reactions are activated, such as the activation of microglia (35, 36) and the infiltration of neutrophils and macrophages in the injured site, which will lead to edema progression, cell death, and permanent nerve injury. We note that with an increase in the quartile of PLR, the platelet count gradually increases and the lymphocyte count decreases. In contrast, excessive platelets may reflect increased cytokine release and platelet activation, leading to a devastating inflammatory response, and the lymphocyte count is inversely associated with inflammation. A lower lymphocyte count increases cardiovascular risk and mortality (37). In addition, more and more evidence shows that high PLR is an independent predictor of atherosclerosis (38), acute renal injury (39), and venous thromboembolism in patients with cancer (40). Studies have also shown that high PLR is associated with an increase in long-term major adverse prognostic events in patients with myocardial infarction (41) and acute pulmonary embolism (42), which undoubtedly increases the mortality of patients with ICH.

It is worth noting that the relationship between PLR and ICH mortality may be attributed to other confounding factors and effect modifiers. Therefore, we conducted a stratified analysis by including age (<65 vs. ≥65 years), sex (female vs. male), atrial fibrillation (no vs. yes), COPD (no vs. yes), hyperlipidemia (no vs. yes), and statin user (no vs. yes), and the results were stable. No interaction was found. However, due to the contingency of multiple tests, our hypothesis still needs to be further confirmed in more studies.

There are some limitations to our study. First, since this is an observational analysis, the possibility of uncontrolled or unknown confounders cannot be eliminated, although the data have been adjusted for various confounding factors. Second, the patients' diagnostic data included in this study were obtained from the database through software. Some patients with cerebral hemorrhage underwent hemorrhage transformation after cerebral infarction, which may bring instability to the research results. Third, we could not extract the volume of ICH, other infection factors, operation type, antibiotic use, admission time after onset of ICH, or nutrition of the patients, which may impact the study. We hope that future studies can carefully consider these factors and increase the strength of the evidence. Fourth, the relationship between PLR and mortality in our study can only be speculated on from retrospective cohort studies and can only prove the correlation, not causality. Finally, our study was conducted using the American MIMIC III database, and further investigation is needed to determine whether the observed results can be extrapolated to other populations. Due to these limitations, it is necessary to further determine the study results through an extensive sample of prospective studies in the future.

## 5. Conclusion

We observed a non-linear relationship between PLR and 90-day mortality for patients with ICH, with an inflection point at 145.54 and a minimal risk at 120.9 to <189.8 of PLR.

## Data availability statement

The data analyzed in this study was obtained from the Medical Information Mart for Intensive Care III (MIMIC-III) database, the following licenses/restrictions apply: to access the files, users must be credentialed users, complete the required training (CITI Data or Specimens Only Research) and sign the data use agreement for the project. Requests to access these datasets should be directed to PhysioNet, https://physionet.org/, doi: 10.13026/C2XW26.

## Ethics statement

The studies involving humans were approved by the Massachusetts Institute of Technology and Beth Israel Deaconess Medical Center, and Jiangxi Provincial People's Hospital Ethics Committee. The studies were conducted in accordance with the local legislation and institutional requirements. Written informed consent for participation was not required from the participants or the participants' legal guardians/next of kin in accordance with the national legislation and institutional requirements.

## Author contributions

MY designed the study. ZX, HZ, AF, ZP, and MY had full access to the raw data and took responsibility for the data's integrity and the accuracy of the data analysis. MY and HZ performed the statistical analysis. MY and ZX drafted the initial manuscript. All authors critically revised the manuscript for important intellectual content and read and approved the final version.
