# Supplementary material for: Association between platelet-lymphocyte ratio and 90-day mortality in patients with intracerebral hemorrhage: data from the MIMIC-III database
Source: Front Neurol. 2023 Oct 9;14:1234252. doi: 10.3389/fneur.2023.1234252 (PMC10591107; doi:10.3389/fneur.2023.1234252)
Supplement: Supplementary file 1 [file Data_Sheet_1.PDF]

eTable 1. Association between PLR and 90-Day mortality in multiple regression model in sensitivity analysis.

Variable	n.total	n.event(%)	Crude	*P* value	Model 1	*P* value	Model 2	*P* value
			HR (95% CI)		HR (95% CI)		HR (95% CI)	
Quartiles								
Q1 (<120.9)	261	88 (33.7)	1(Ref)		1(Ref)		1(Ref)	
Q2 (120.9 to <189.8)	260	66 (25.4)	0.70(0.51~0.96)	0.026	0.67 (0.48~0.93)	0.016	0.65 (0.47~0.9)	0.01
Q3 (189.8 to <296.5)	261	71 (27.2)	0.74 (0.54~1.02)	0.063	0.71 (0.52~0.98)	0.036	0.65 (0.47~0.9)	0.009
Q4 (≥296.5)	261	86 (33)	0.94 (0.70~1.27)	0.688	0.96 (0.7~1.31)	0.788	0.87 (0.63~1.18)	0.36
P for trend. test				0.795		0.885		0.410
Categories								
Q1 (<120.9)	261	88 (33.7)	1.39 (1.06~1.82)	0.016	1.45 (1.1~1.91)	0.008	1.54 (1.17~2.03)	0.002
Q2-3(120.9 to <296.5)	521	137 (26.3)	1(Ref)		1(Ref)		1(Ref)	
Q4 (≥296.5)	261	86 (33)	1.31 (1.00~1.71)	0.052	1.39 (1.05~1.83)	0.020	1.33 (1.01~1.76)	0.045
P for trend. test				0.693		0.793		0.383

Model 1: Adjusted for sex, age, COPD, Heart Failure, Hyperlipemia, Ethnic, Blood glucose,Serum creatinine, Serum urea nitrogen, Serum calcium, APTT, Alcohol drinker, Location of ICH, Statin User, Antiplatelet agents, hypertension history, mean arterial pressure, and anticoagulant use.

Model 2: Adjusted for the variables in Model 1 plus the blood transfusion, sepsis and the use of mechanical ventilation.
